# Transcranial direct current stimulation of the occipital lobes with adjunct lithium attenuates the progression of cognitive impairment in patients with first episode schizophrenia

**DOI:** 10.3389/fpsyt.2022.962918

**Published:** 2022-09-13

**Authors:** Chuanjun Zhuo, Hongjun Tian, Chunhua Zhou, Yun Sun, Xinying Chen, Ranli Li, Jiayue Chen, Lei Yang, Qianchen Li, Qiuyu Zhang, Yong Xu, Xueqin Song

**Affiliations:** ^1^Key Laboratory of Real Time Brain Circuit Tracing in Neurology and Psychiatry (RTBNP_Lab), Tianjin Fourth Center Hospital, Tianjin Fourth Central Hospital of Tianjin Medical University, Tianjin, China; ^2^Key Laboratory of Multiple Organ Damages of Major Psychoses (MODMP_Lab), Tianjin Fourth Center Hospital, Tianjin Medical Affiliated Tianjin Fourth Central Hospital, Nankai University Affiliated Tianjin Fourth Center Hospital, Tianjin, China; ^3^Henan Psychiatric Transformation Research Key Laboratory, Zhengzhou University, Zhengzhou, Henan, China; ^4^Biological Psychiatry International Joint Laboratory of Henan, Zhengzhou University, Zhengzhou, Henan, China; ^5^Department of Pharmacology, The First Hospital of Hebei Medical University, Shijiazhuang, Hebei, China; ^6^t-DCS and r-TMS Center of Tianjin Anding Hospital, Tianjin Mental Health Center of Tianjin Medical University, Tianjin, China; ^7^Department of Psychiatry, The First Hospital Affiliated to Shanxi Medical University, Taiyuan, China; ^8^Department of Psychiatry, The First Affiliated Hospital of Zhengzhou University, Zhengzhou, Henan, China

**Keywords:** schizophrenia, cognitive impairment, occipital lobe, t-DCS, r-TMS, lithium

## Abstract

**Background:**

There is no standard effective treatment for schizophrenia-associated cognitive impairment. Efforts to use non-invasive brain stimulation for this purpose have been focused mostly on the frontal cortex, with little attention being given to the occipital lobe.

**Materials and methods:**

We compared the effects of nine intervention strategies on cognitive performance in psychometric measures and brain connectivity measured obtained from functional magnetic resonance imaging analyses. The strategies consisted of transcranial direct current stimulation (t-DCS) or repetitive transcranial magnetic stimulation (r-TMS) of the frontal lobe or of the occipital alone or with adjunct lithium, or lithium monotherapy. We measured global functional connectivity density (gFCD) voxel-wise.

**Results:**

Although all nine patient groups showed significant improvements in global disability scores (GDSs) following the intervention period (vs. before), the greatest improvement in GDS was observed for the group that received occipital lobe-targeted t-DCS with adjunct lithium therapy. tDCS of the occipital lobe improved gFCD throughout the brain, including in the frontal lobes, whereas stimulation of the frontal lobes had less far-reaching benefits on gFCD in the brain. Adverse secondary effects (ASEs) such as heading, dizziness, and nausea, were commonly experienced by patients treated with t-DCS and r-TMS, with or without lithium, whereas ASEs were rare with lithium alone.

**Conclusion:**

The most effective treatment strategy for impacting cognitive impairment and brain communication was t-DCS stimulation of the occipital lobe with adjunct lithium therapy, though patients often experienced headache with dizziness and nausea after treatment sessions.

## Introduction

Although antipsychotic agents can alleviate positive and negative symptoms of schizophrenia, there is not yet a standard supported intervention for addressing the additional common core symptom of cognitive impairment, which is present in some 80% of patients with schizophrenia and highly impactful on prognosis (1). There has been evidence of some positive, albeit mostly delayed, effects of non-invasive treatments, such as transcranial direct current stimulation (t-DCS), repeated transcranial magnetic stimulation (r-TMS), vagus nerve stimulation, and deep brain stimulation on cognitive performance in psychiatric patients (2–4). Among them, t-DCS appears to be particularly promising for improving attention/vigilance, while both t-DCS and r-TMS may produce small improvements in working memory (5). There remains an ongoing need to explore and identify methods that may improve cognitive impairments in schizophrenic patients, including potential pharmacotherapeutic approaches, such as TAK-071, brain-derived neurotrophic factor, and N-acetylaspartic acid, which are currently being tested in animal studies (1, 6–10).

For three decades, the occipital lobe has been suggested to be potentially important in schizophrenia-associated cognitive impairments, though this possibility has received little research attention (11–14). Functionally, the occipital lobe is known to play a pivotal role in cognition-related information processing (15–18). Posterior brain regions, including the superior parietal-occipital cortex, have been shown to become activated together with the dorsal premotor cortex in tasks requiring reaching and complex visually guided grasping movements (19). Roelfsema and de Lange (20) posited that the primary visual cortex might serve as a multi-scale cognitive blackboard (21), while others have published evidence consistent with the possibility that the visual cortex, especially the portion contained in the occipital lobe, may play a pivotal role in cognitive adaption (22–25).

Notwithstanding, research focused on addressing cognitive impairments tends to be heavily focused on the frontal lobe, especially the prefrontal cortex. Briefly, in the last two decades, there has been a convergence of studies demonstrating involvement of the visual cortex in cognitive processing. Simultaneously, t-DCS of the occipital lobe has been shown to influence cognitive processing and to have benefits from some neuropsychiatric conditions. Based on these observations, we examined the effects of t-DCS of the occipital lobe on functional activity and, subsequently, on cognitive impairment in patients with schizophrenia.

Given that the skull in the posterior cranium is thinner than that in the frontal cranium, stimulating the occipital lobe would be expected to be easier than stimulating the frontal lobe, though this accessibility may also be accompanied by an increased risk of adverse secondary effects (ASEs). Based on suggestions in the above-mentioned literature, we designed a study to examine whether stimulation of the posterior brain may be effective for alleviating schizophrenia-related cognitive impairments while maintaining an acceptable level of risk. Here, we report a pilot study in which we tested the relative efficacies of nine strategies for improving cognitive function in first-episode patients with schizophrenia. The strategies involve use of a non-invasive stimulation treatment modality (t-DCS or r-TMS), use of lithium (a neuroprotective agent shown to improve mild cognitive impairment) (26), or use of a combination of each mode of stimulation with lithium. Stimulation was applied to the occipital lobe or to the frontal lobe. Hence, the nine strategy treatment groups were as follows: occipital lobe t-DCS with adjunct lithium (O-tDCS + Li); occipital lobe r-TMS with adjunct lithium (O-rTMS + Li); lithium monotherapy (Li); occipital lobe t-DCS monotherapy (O-tDCS); occipital lobe r-TMS monotherapy (O-rTMS), frontal lobe t-DCS with adjunct lithium (F-tDCS + Li); frontal lobe r-TMS with adjunct lithium (F-rTMS + Li); t-DCS monotherapy (F-tDCS); and frontal lobe r-TMS monotherapy (F-rTMS). Intervention efficacy was determined based on psychometric evaluations and analyses of global functional connectivity density (gFCD) values obtained by magnetic resonance imaging (MRI).

## Materials and methods

### Subjects

We recruited patients who were being treated for their first episode of schizophrenia in the department of Psychiatry at Tianjin Fourth Center Hospital or Zhengzhou University hospital. The inclusion criteria were: (1) schizophrenia diagnosis in accordance with the criteria of the Diagnostic and Statistical Manual of Mental Disorders, Fourth Edition (DSM-IV) (27); (2) first episode, illness duration ≤6 months; (3) stable at the time of study participation; (3) no additional mental disorder beyond schizophrenia in one’s life history based on application of the Structured Clinical Interview for the DSM-IV non-patient edition (28) by two experienced clinical psychiatrists who came to a consensus for each case; (4) no severe physical illness or neurological comorbidities that can influence the study; (5) no contraindication to MRI; and (6) presenting with evidence of a cognitive impairment evidenced by a MATRICS Consensus Cognitive Battery (MCCB) (29) score lower than the average for China and an MCCB-based Global Deficit Score (GDS) (30) ≥3. The drug exposure of each patient in the week preceding each MRI scan was converted to chlorpromazine equivalent dosage.

The Medical Research Ethics Committee of Tianjin Fourth Center Hospital approved this study. Written informed consent was obtained after each prospective participant with a complete description of the study.

### Psychometric assessments

Schizophrenia symptom severity was evaluated with the Positive and Negative Syndrome Scale (PANSS) (31). Cognitive impairment was determined with the MCCB and (MCCB-based) GDS.

### Transcranial direct current stimulation

For occipital lobe t-DCS, a 2-mA current was delivered with the anode and cathode placed over the right and left occipital lobe, respectively. For frontal lobe t-DCS, a 2-mA current was delivered with the anode and cathode placed over the right and left dorsal lateral prefrontal lobe dorsal lateral prefrontal lobe, respectively. In both interventions, current was delivered for a total of 20 min in each session. The full intervention was 72 sessions (completed within 24 weeks).

### Repetitive transcranial magnetic stimulation

In this study, 10-Hz r-TMS was presented at 110% of the motor threshold. Each stimulation lasted for 4 s with 26-s intervals; a total of 1,600 pulses were delivered within each 20-min daily session. According to group, r-TMS was delivered to the left occipital lobe or prefrontal cortex, with the stimulation site 5.5 cm anterior to the optimal site in each case on a para-sagittal plane. The full intervention was 72 sessions (completed within 24 weeks).

### Lithium

Lithium exposure was maintained in the range of 0.4–0.8 mmol/L. Blood concentration of lithium was monitored biweekly.

### Magnetic resonance imaging data acquisition

Participants were subjected to structural MRI and functional MRI (fMRI) pre- and postintervention. Imaging data were obtained with a 3.0-T Discovery MR750 system (General Electric, Milwaukee, WI). The participants wore closely fitted foam padding to minimize their head movements, and they wore earplugs to reduce exposure to scanner-generated noise. We used a brain volume sequence with the following parameters to obtain three-dimensional sagittal T1-weighted images: repetition time (TR)/echo time (TE)/inversion time (TI) = 8.2 ms/3.2 ms/450 ms; flip angle, 12°; field of view/matrix = 256 × 256 mm/256 × 256; and 1-mm slice thickness without gaps yielding 188 sagittal slices. We used a gradient-echo single-short echo planar imaging sequence with the following parameters to obtain resting state fMRI data: TR/TE = 2000/45 ms; field of view/matrix = 220 × 220 mm/64 × 64; flip angle, 90°; and 4-mm slice thickness with 0.5-mm gaps yielding 32 interleaved transverse slices and 180 volumes. While being prepared for scanning, each participant was asked to relax with their eyes closed, to think of nothing in particular, and to move as little as they can but not fall asleep during scanning.

### Functional magnetic resonance imaging data preparation

The fMRI data were preprocessed in SPM8 software, available at www.fil.ion.ucl.ac.uk/spm. The first 10 volumes of each scan were discarded under the presumption that participants may have been still adapting and the system may have still been reaching equilibrium. The volumes obtained thereafter were then corrected for inter-slice acquisition time delays and then realigned to correct for motion between slices. Ultimately, all of the fMRI data collected were confirmed to be within predefined translational/rotational motion thresholds (< 2 mm/ < 2°). Frame-wise displacement did not differ significantly (*t* = 0.38, *P* = 0.26) between postintervention (0.145 ± 0.014) and preintervention baseline (0.120 ± 0.010) scans. We regressed nuisance covariates (i.e., six motion parameters, their first-time derivations, and average ventricular space and white matter blood-oxygen-level-dependent signals) out of the data. Because signal spiking caused by head motion can contaminate fMRI results despite regressing out of linear motion parameters (32), we also regressed out spike volumes of specific volumes with a frame-wise displacement > 0.5. The datasets were band-pass filtered (0.01–0.08 Hz). For normalization, each structural MR image was co-registered linearly relative to the mean functional image and co-registered to Montreal Neurological Institute (MNI) space. Employing co-registration parameters, we spatially normalized each filtered functional volume to MNI space and resampled it into a 3-mm cubic voxel.

### Global functional connectivity density

We assessed gFCD as an index of functional connectivity alterations throughout the brain. We used Tomasi and Volkow’s (33) method in a Linux platform to calculate the gFCD of each voxel (Totally 57777 voxels in the brain was defined in the present study). Inter-voxel functional connectivity was determined with Pearson’s linear correlation analyses, applying a significance criterion of *R* > 0.6 [previously shown to be the optimal threshold by Tomasi and Volkow (33)]. We determined gFCDs within the cerebral gray matter mask. The total number of functional connections that a given voxel had with all other voxels was taken as the gFCD of that voxel. This calculation was repeated for each voxel in the fMRI scan dataset. To maximize distribution normality, a grand mean gFCD was obtained by dividing by the mean gFCD value of all of the imaged brain voxels within each patient’s scan dataset. The gFCD maps obtained were subjected to spatial smoothing with a 6 × 6 × 6-mm full-width at half maximum Gaussian kernel. To check for effects of correlation threshold selection on the gFCD analysis, we calculated gFCD maps on two additional thresholds, namely *R* > 0.2 and R > 0.4.

### Grave matter volume

We used voxel-based morphometry to calculate GMVs voxel-wise in VBM8 software.^1^ Employing the standard segmentation model, we segmented structural MRIs into gray matter, white matter, and cerebrospinal fluid components. We completed an initial affine registration of the gray matter concentration map into MNI space. Then, using diffeomorphic anatomical registration through the exponentiated Lie algebra technique, we warped the gray matter concentration images non-linearly; the results obtained were resampled to a voxel size of 3 × 3 × 3 mm. Then, to obtain the relative GMV of each voxel, the gray matter concentration maps were multiplied by non-linear determinants derived from the aforementioned spatial normalization. The GMV images were smoothed with a Gaussian kernel of 6 × 6 × 6-mm full-width at half maximum prior to being subjected to statistical analyses.

### Statistical analysis

Means are reported with standard errors of the mean. Using a general linear model with age and sex as nuisance variables and a permutation-based inference tool for non-parametric statistics in the FMRIB diffusion toolbox (FSL 4.0, available at www.fmrib.ox.ac.uk/fsl), gFCD values were compared voxel-wise with 5000 permutations. Differences with a *p* value less than 0.05, after family wise error correction by threshold-free cluster enhancement, were considered statistically significant. Group comparisons of gFCD were run with GMV as an additional covariate of no interest at the voxel-wise level. These analyses were conducted for data obtained applying thresholds of R > 2, R > 3 (main analysis), and R > 4. For each cluster that met the significance threshold, we extracted the mean gFCD value of that cluster for each subject. Partial correlation coefficients were calculated to detect associations between gFCD and clinical variables (i.e., chlorpromazine equivalent dosage, illness duration, and psychometric scores). Age and gender effects were controlled. Multiple comparisons were corrected for with Bonferroni’s method (threshold *p* < 0.05). Correlation analyses between gFCD and clinical variables were performed for the whole brain voxel-wise. Correlational analyses were conducted with a linear regression model with age and gender as covariates of no interest. Multiple comparisons were corrected for according to the family wise error method (*p* < 0.05).

## Results

### Sample

A total of 450 right-handed participants were enrolled and placed into the nine intervention groups. Among them, 383 patients completed the study (85.11% completion rate).

### Comparison of the effects of nine treatment strategies on cognitive assessment scores

Cognitive assessment scores before and after the 24-week intervention period are compared among the intervention groups in Table 1. Before the interventions, the groups had similar GDSs. All nine interventions had significant positive effects on GDS and there was a significant effect of group on GDS. The largest improvements in GDS were observed for the O-tDCS + Li group (mean increase, 1.81 ± 0.12, *p* < *0.05*), O-rTMS + Li group (mean increase 1.24 ± 0.22, *p* < 0.05), and Li group (mean increase, 1.04 ± 0.03, *p* < 0.05). Thereafter, in order of next best to least impactful treatment strategies (all *p* < 0.05), the remaining groups performed as follows: O-tDCS; O-rTMS; F-tDCS + Li; F-rTMS + Li; F-tDCS; and F-rTMS.

**TABLE 1:** Effects of 24-week treatments on cognitive impairment.

Variable	O-tDCS + Li	O-rTMS + Li	Li	O-tDCS	O-rTMS	F-tDCS + Li	F-rTMS + Li	F-tDCS	F-rTMS	ANOVA *P*
*Pre-intervention*										
MCCB domain scores										
SP	35.30 ± 5.45	35.14 ± 4.25	34.07 ± 2.30	30.28 ± 3.60	32.87 ± 2.35	36.02 ± 1.82	34.70 ± 1.13	33.47 ± 1.05	34.55 ± 10.86	0.037
AV	31.47 ± 2.62	34.55 ± 3.12	34.33 ± 1.36	31.36 ± 1.00	33.32 ± 1.42	32.16 ± 1.28	34.15 ± 0.45	32.20 ± 1.63	35.06 ± 2.13	0.012
WM	31.50 ± 1.30	30.57 ± 1.39	34.22 ± 3.20	30.33 ± 4.75	32.11 ± 1.87	30.25 ± 1.52	30.02 ± 0.45	30.51 ± 0.69	31.29 ± 1.55	0.041
VerbL	30.20 ± 2.77	30.62 ± 1.24	32.11 ± 1.33	30.50 ± 1.44	31.25 ± 1.08	32.44 ± 1.45	32.53 ± 1.09	31.44 ± 1.22	30.96 ± 1.59	0.053
VisL	30.22 ± 2.23	32.09 ± 1.12	31.03 ± 1.80	30.21 ± 1.45	30.88 ± 2.00	30.25 ± 1.44	30.55 ± 0.85	30.28 ± 3.78	33.25 ± 0.58	0.029
Reas	32.25 ± 2.02	31.24 ± 2.12	30.44 ± 3.09	30.00 ± 1.36	34.22 ± 1.45	30.04 ± 1.85	30.00 ± 0.77	31.25 ± 0.80	32.14 ± 1.55	0.049
SR	31.20 ± 2.36	32.15 ± 1.33	32.03 ± 1.42	31.72 ± 1.25	32.02 ± 1.28	32.11 ± 1.28	32.40 ± 1.25	32.55 ± 0.98	34.00 ± 1.41	0.047
GDS	3.54 ± 1.54	3.50 ± 1.20	3.20 ± 2.14	3.14 ± 1.45	3.65 ± 0.54	3.47 ± 1.26	3.66 ± 0.96	3.47 ± 1.02	3.02 ± 0.96	3.52 ± 1.36
*Post-intervention*										
SP	27.33 ± 1.42	21.65 ± 1.62	25.23 ± 1.36	25.00 ± 1.25	23.44 ± 0.63	26.82 ± 1.40	25.36 ± 0.58	25.63 ± 1.42	22.33 ± 2.36	0.046
AV	25.22 ± 1.25	23.42 ± 1.02	22.43 ± 1.45	21.44 ± 1.23	23.02 ± 1.26	22.16 ± 2.20	20.03 ± 0.78	22.14 ± 0.58	23.25 ± 1.25	0.039
WM	26.14 ± 0.63	23.54 ± 1.45	21.25 ± 1.25	22.45 ± 1.10	21.20 ± 0.87	21.22 ± 1.02	21.36 ± 1.56	21.89 ± 1.25	21.29 ± 0.69	0.021
VerbL	25.23 ± 1.25	24.35 ± 1.00	21.23 ± 1.44	20.33 ± 2.30	23.02 ± 0.85	20.48 ± 0.69	21.53 ± 0.64	21.36 ± 0.89	21.66 ± 1.23	0.028
VisL	24.36 ± 1.56	23.35 ± 0.36	21.36 ± 0.87	23.69 ± 1.23	24.78 ± 1.56	23.69 ± 1.35	21.36 ± 1.23	23.36 ± 1.28	19.28 ± 1.36	0.003
Reas	26.25 ± 1.36	25.64 ± 2.02	22.15 ± 1.09	21.00 ± 1.00	18.82 ± 1.25	17.66 ± 1.83	18.50 ± 1.55	18.25 ± 1.36	18.20 ± 2.20	0.001
SR	25.33 ± 0.89	24.22 ± 1.88	22.07 ± 0.74	20.36 ± 1.28	19.12 ± 2.23	18.56 ± 0.98	19.25 ± 2.32	18.99 ± 2.10	17.69 ± 0.69	0.001
GDS	2.56 ± 0.89	2.62 ± 1.26	2.77 ± 1.26	2.65 ± 1.10	2.82 ± 1.10	2.96 ± 1.40	2.83 ± 2.12	2.99 ± 1.29	2.36 ± 0.58	0.024
Reduction in GDS,%	33.62	24.24	22.75	23.33	18.57	17.23	10.22	8.11	6.63	4.56

O-, occipital lobe targeted; t-DCS transcranial direct current stimulation; + Li, with adjunct lithium; rTMS, repetitive transcranial magnetic stimulation; Li, lithium monotherapy; F-, frontal lobe targeted; ANOVA, analysis of variance; MCCB, MATRICS Consensus Cognitive Battery; GDS, Global Disability Score; SP, speed processing; AV, attention vigilance; WM, working memory; VerbL, verbal learning; VisL, visual learning; Reas, reasoning; SR, social recognition.

### Comparison of the effects of nine treatment strategies on global functional connectivity density

**FIGURE 1:**
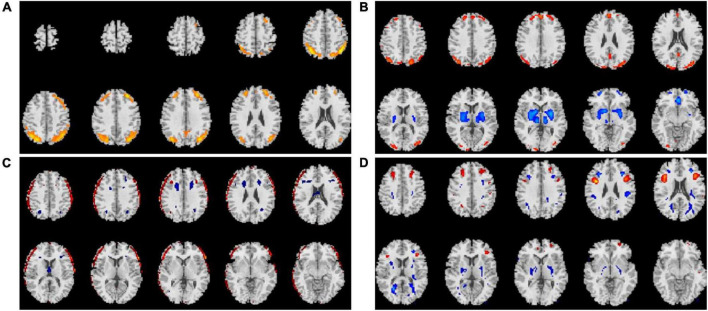
Comparison of gFCDs before versus after 24-week treatments inclusive of f-DCS. **(A)** O-tDCS + Li group. **(B)** O-tDCS group. **(C)** F-fDCS + Li group. **(D)** F-tDCS group.

**FIGURE 2:**
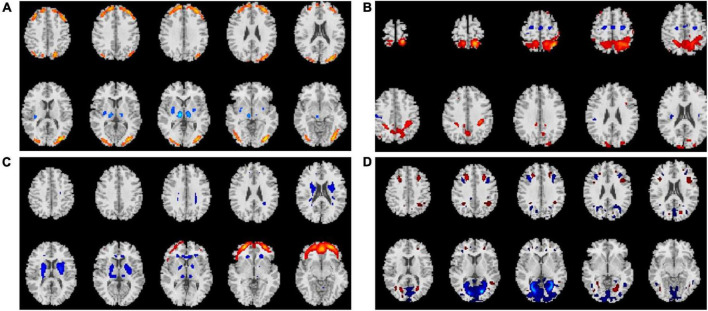
Comparison of gFCDs before versus after 24-week treatments inclusive of r-TMS. **(A)** O-rTMS + Li group. **(B)** O-rTMS group. **(C)** F-rTMS + Li group. **(D)**. F-rTMS group.

**FIGURE 3:**
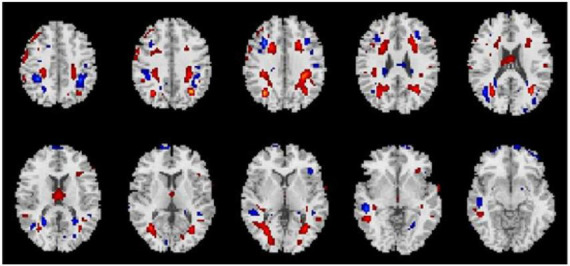
Comparison of gFCDs before versus after 24-week treatment with only lithium (Li group).

Average changes in gFCD before versus after each intervention are shown in Figures 1–3. Most remarkably, we found that whole-brain gFCD increased 72-fold in the O-tDCS + Li group. Thereafter, in order of next most marked change to least change, the remaining groups performed as follows: O-rTMS + Li; Li; O-tDCS; O-rTMS; F-tDCS + Li; F-rTMS + Li; F-tDCS; and F-rTMS. This rank order is consistent with the order of impact we observed above for GDS.

### Adverse secondary effects

Substantial portions of the patients in all of the t-DCS and r-TMS treatment groups, with or without adjunct lithium, experienced ASEs. In the Li group, there was only a single patient who reported experiencing an ASE, namely fatty diarrhea (0.2%, 1/50). The most common ASEs for each other group, besides the Li group, were as follows: O-tDCS + Li, headache with dizziness and nausea (75.0%, 24/32); O-rTMS + Li, dizziness and nausea (73.80%, 31/42); O-tDCS, headache (79.49%, 31/39); O-rTMS, dizziness (alone) (78.57%, 33/42); F-tDCS + Li, moderate headache with dizziness (77.5%, 31/40); O-rTMS + Li, mild headache and nausea (57.5%, 23/40); F-tDCS, moderate headache (52.08%, 25/48); F-tDCS, mild headache (48.00%, 24/50).

## Discussion

To the best of our knowledge, this study was the first to compare the effects of multiple new cognitive treatment strategies for schizophrenia-associated cognitive impairment, inclusive of targeting the occipital lobe, with two traditional strategies (i.e., dorsal lateral prefrontal lobe stimulation monotherapies). Our data demonstrated that stimulation of the occipital lobe with t-DCS combined with adjunct lithium treatment was beneficial for mitigating the progression of worsening in schizophrenic patients’ cognitive performance. However, this treatment strategy was commonly associated with dizziness and nausea.

Our analysis of gFCD, an index of whole brain connectivity, demonstrated that cognitive impairments were alleviated by r-TMS stimulation with adjunct lithium as well as by lithium monotherapy. Because lithium monotherapy did not produce ASEs frequently, while the other treatment strategies produced ASEs did, lithium monotherapy may be better accepted by patients, especially in the long term.

The present data support the notion that the posterior brain may play an important role in the cognitive impairment experienced by patients with schizophrenia. Furthermore, these data also demonstrated that brain connectivity alterations produced in response to occipital lobe stimulation can improve the cognitive impairment. Interestingly, we observed that t-DCS of the occipital lobe increased gFCD in the occipital lobe as well as in the frontal lobe, suggesting that occipital lobe targeted t-DCS can enhance whole brain connectivity, which may underlie its efficacy for improving cognitive impairment.

The present finding of lithium treatment-induced enhancement of whole-brain functional connectivity is consistent with previous studies reporting that lithium can improve the cognitive impairment in patients diagnosed with mild cognitive impairment or dementia (34, 35). Implicated mechanisms for these lithium benefits include inhibition of glycogen synthase kinase-3 and reduction of beta-amyloid and hyper-phosphorylated tau (34, 35). Our data, from a large-scale imaging perspective, provide evidence suggesting that lithium can improve cognitive impairments by enhancing functional connectivity on a whole-brain level. Functional connectivity represents the information communication capability of the brain (36–42). Thus, increased gFCD, especially in the occipital lobe, basal ganglia, and frontal lobe, reflects enhanced communication throughout the brain. Further research is needed to explore whether lithium indeed mitigates cognitive impairment by enabling better brain-wide communication (43–46).

The present data showed that gFCD in occipital regions was reduced by t-DCS with adjunct lithium but not by r-TMS with adjunct lithium. It is possible, that the t-DCS stimulus is more powerful than the r-TMS stimulus, though we cannot make any conclusions regarding the reason for this difference. We did observe lesser gFCD values in the hippocampus following r-TMS with lithium treatment than following t-DCS with lithium treatment, which could reflect a relatively weaker effect of the former.

Notably, we observed that lithium monotherapy resulted in increased gFCD values in the temporal lobe and posterior parietal lobe compared with pre-treatment values. It is well known that the posterior parietal lobe is important for cognitive ability. Moreover, lithium has been shown to enhance cognitive performance in a temporal-lobe dependent visual task. Thus, we postulate that lithium may support cognitive performance by way of its positive effects on functional connectivity in the temporal and posterior parietal lobes, at least in part. Although occipital lobe-targeted t-DCS monotherapy induced gFCD increases in the occipital lobe and prefrontal cortex in this study, it did not increase gFCD in the basal ganglia region. Conversely, when lithium was given alone or as an adjunct with t-DCS, gFCD increases in the basal ganglia region were seen.

Occipital lobe treatment with t-DCS seemed to be more beneficial in terms of cognitive performance measures than r-TMS. This difference might be reflected in the ability of the former to increase the gFCD in basal ganglia regions and the prefrontal cortex. Although frontal lobe-targeted t-DCS with adjunct lithium resulted in increased gFCD in the frontal, temporal, and parietal lobes, it did not increase gFCD in posterior brain regions. Thus, it appears that targeting of t-DCS to the occipital lobe may have more distal connectivity benefits than targeting t-DCS to the frontal lobe. Similarly, frontal lobe-targeted r-TMS increased gFCD alterations in the prefrontal cortex without augmenting gFCD in the basal ganglia region or in posterior brain regions, suggesting that t-DCS stimulation of the frontal lobe may enhance brain activity in only the frontal lobe.

### Limitations

There are several limitations in this study. First, the patient sample was heterogenous with respect to cognitive impairment, illness duration, and symptom severity. We used covariance analysis to regress out these differences in our gFCD analysis and we compared change in GDS (rather than absolute scores) to compare effects across the nine strategy groups. However, this method cannot assure all possible downstream influences of these differences are eliminated. In a future larger study, we will enroll sufficient numbers of patients to balance out these differences as much as possible. Second, we focused on comparing patients before versus after treatment, but we did not have an untreated control group, which limited the amount of information we could obtain. Third, it is unclear why no correlations between gFCD alteration and GDS alteration were found for any of the treatment strategy groups. We hope that larger sample studies will clarify this question.

## Conclusion

Of the nine treatment strategies compared in this study, t-DCS stimulation of the occipital lobe with adjunct lithium therapy had the strongest beneficial effect on cognitive impairments, although patients in this group commonly experienced headache with dizziness and nausea.

## Data availability statement

The datasets generated and analyzed during this present study are available from the corresponding authors upon reasonable request.

## Ethics statement

The studies involving human participants were reviewed and approved by the Ethics Committee of Tianjin Fourth Center Hospital. The patients/participants provided their written informed consent to participate in this study.

## Author contributions

CJZ, HT, and CHZ contributed to the conception and writing of the manuscript. YS, XC, RL, JC, LY, QL, QZ, YX, and XS contributed to the article and approved the submitted version. All authors contributed to the article and approved the submitted version.

## Footnotes

1
http://dbm.neuro.uni-jena.de/vbm8

## Abbreviations

t-DCS: direct current stimulation
r-TMS: Repeated transcranial magnetic stimulation
ASEs: Adverse secondary effects
Li: Lithium
MRI: Magnetic resonance imaging
DSM-IV, The Diagnostic and Statistical Manual of Mental Disorders: Fourth Edition
MCCB: The MATRICS Consensus Cognitive Battery
GDS: Global Deficit Score
PANSS: Positive and Negative Syndrome Scale
fMRI: Functional magnetic resonance imaging
gFCD: Global functional connectivity density
GMV: Grave matter volume.
